# Multi-task meta-attention network for traditional Chinese medicine diagnostic recommendation

**DOI:** 10.3389/fpubh.2025.1549679

**Published:** 2025-08-01

**Authors:** YingShuai Wang, YanLi Wan, HongPu Hu

**Affiliations:** ^1^Institute of Medical Information/Library, Chinese Academy of Medical Sciences and Peking Union Medical College, Beijing, China; ^2^School of Marxism, School of Humanities and Social Sciences, Chinese Academy of Medical Sciences and Peking Union Medical College, Beijing, China

**Keywords:** deep learning, feature engineering, recommendation technology, traditional Chinese medicine, clinical decision support, smart healthcare

## Abstract

**Background:**

With the continuous growth of medical data and advancements in medical technology, there is an increasing need for personalized and accurate assisted diagnosis. However, implementing recommendation systems in healthcare presents numerous challenges, requiring further in-depth research.

**Objective:**

This study explores the application of recommendation technology in smart healthcare. The primary goal is to design a deep learning model that effectively integrates medical knowledge for improved diagnostic support.

**Methods:**

We first developed a feature engineering process tailored to the characteristics and requirements of medical data. This process involved data preparation, feature selection and transformation to extract informative features. Subsequently, a knowledge-matching deep learning model was designed to analyze and predict medical data. This model enhances evaluation metrics through its capabilities in nonlinear fitting and feature learning.

**Results:**

Experimental results indicate that our proposed deep learning model achieves an average improvement of +2.7% in the core metrics Hits@10 compared to baseline models in the Traditional Chinese Medicine (TCM) clinical recommendation scenario. The model effectively processes medical data, providing accurate predictions and valuable insights to support clinical decision-making.

**Conclusion:**

This research provides significant support for the advancement and application of smart medical technology. Our deep learning model demonstrates strong potential for medical data analysis and clinical decision-making. It can contribute to enhanced healthcare quality and efficiency, ultimately advancing the medical field.

## 1 Introduction

With the advancements in big data and artificial intelligence technologies, smart healthcare has gradually emerged as a new trend. Recommendation technology, with its strengths in personalized information delivery and decision support, offers new possibilities for the advancement of smart healthcare. By analyzing medical and health data, recommendation technology can provide personalized advice and services to both doctors and patients, thereby enhancing the accuracy and efficiency of medical decision-making. Xiyu Shen (1) applied a deep learning-based hierarchical attention network to construct models for doctors and patients using consultation records. This approach strengthened the interaction between the doctor and patient vectors, assigned higher weights to doctors and patients with similar conditions, and calculated the doctor’s recommendation value. Yinghua Wu (2) explored the application scenarios of ChatGPT and other next-generation AI technologies in smart healthcare, including areas such as medical management, medical technology for doctors, and patient healthcare. Quan Chen (3) investigated the application of ChatGPT and other next-generation AI technologies in smart healthcare. He developed a transfer relationship model between abnormal signs and drugs based on a graph neural network and integrated abnormal sign information to enable accurate drug recommendations. Recommendation systems in the medical and healthcare fields are continuously evolving, encompassing areas such as medication recommendations, prescription suggestions, assisted diagnosis, precision treatment, and health monitoring. Xiaojing Hu (4) designed a research questionnaire to assess patients’ willingness to recommend, using the net recommendation value, and employed multivariate logistic regression analysis to explore the factors influencing patients’ willingness to recommend. Fang Wang (5) analyzed the current state of health management services both domestically and internationally, and explored pathways for constructing intelligent prescriptions based on disease risk. Pinsky (6) analyzes the characteristics of standard datasets and pre-trained models in the medical field, integrates them with practical applications of artificial intelligence, and explores the requirements for large model development to promote the innovative advancement of medicine. Large Language Models (LLM) have archived initial success in medicine. Liu et al. used pre-training and fine-tuning to create a large language model-based diagnostic system, to assist physicians in formulating diagnosis and treatment plans (7). Li et al. developed a system that uses deep learning and language models to aid diabetes care and retinopathy screening, improving patient follow-up (8).

Although recommendation technology and machine learning demonstrate great potential in the field of smart healthcare, they still face numerous challenges. Firstly, the quality and scale of healthcare data are inconsistent, and the data often exhibit heterogeneity and incompleteness, which complicates model construction. Additionally, existing machine learning have limitations in medical applications, such as a lack of sufficient interpret ability, difficulty in addressing the diversity of clinical scenarios, inadequate generalization ability, and the need for improved reliability and stability in actual clinical decision-making. The unique characteristics of medical field also pose numerous challenges for the development of large language models, including the issue of hallucinations generating inaccurate information, high deployment costs to ensure precision, lack of currency making it difficult to reflect the latest medical advancements, bias and toxicity potentially leading to unfair treatment recommendations, and privacy and security concerns when handling personal health information. The aim of this study is to conduct an in-depth exploration of the application of recommendation technology in smart healthcare, with the goal of providing theoretical guidance and technical support to advance the development of smart healthcare, and to further enhance the intelligence and humanization of healthcare services.

**Figure 1:**
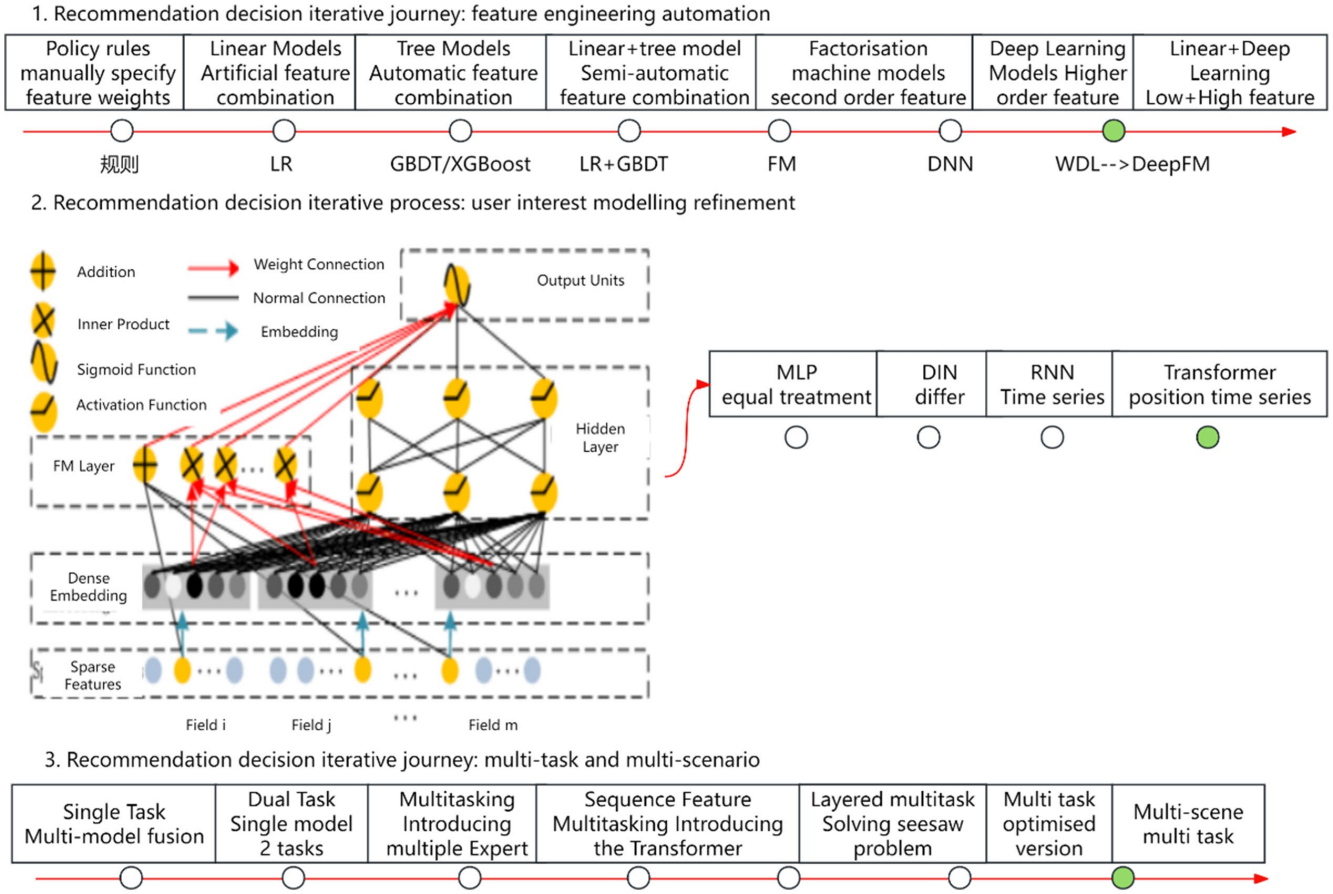
Recommendation algorithm evolutionary paradigms. Author: Yingshuai Wang, Date: 2024-12-21.

Recommendation algorithms have evolved along three primary paradigms, as illustrated in Figure 1. The first paradigm involves the automation of feature engineering, beginning with manually defined feature weights and strategy rules, progressing through logistic regression (LR) (9), gradient boosting decision trees (GBDT) (10, 11), and factorization machines (FM) (12). In recent years, deep neural networks (DNN) (13) have become dominant, owing to their remarkable expressiveness and flexibility. Models such as Wide & Deep (W&D) (14) exemplify this evolution by effectively combining low-order and high-order features. The second paradigm focuses on refining user interest modeling. It begins with the Multi-Layer Perceptron (MLP) (15), where user interests are treated uniformly. This is followed by the Deep Interest Network (DIN) (16), which differentiates user interests, and culminates in the Transformer model (17), incorporating both location and temporal sequences. The third paradigm centers on multi-task, multi-scenario modeling. It evolved from single-task, multi-model fusion to multi-task learning with the introduction of multiple Experts (18), then to sequence-based multi-tasking using the Transformer, and further to multi-layer multi-tasking. Ultimately, it leads to multi-scenario, cross-domain recommendation.

Personalized recommendation is a feasible solution for smart healthcare. However, existing recommendation technology is primarily applied in the commercial sector, whereas recommendation systems in the medical field differ significantly in terms of objectives, scenarios, and data. For instance, e-commerce recommendations are based on user purchase history to establish relationships between users and products, focusing on building user profiles and product profiles. In medical scenarios, however, patient evaluations or doctor’s consultation information are typically used for modeling, and the data scale is relatively small. As a result, commodity recommendation models cannot be directly applied, presenting unique challenges and opportunities for exploration in modeling and design. Recommendation research in the medical field primarily relies on shallow deep learning techniques for model construction, with recommendation strategies largely based on statistical methods or machine learning mechanisms. These approaches typically use a single method for interactive vector calculations. Drawing from the modeling approach used in deep learning for commodity recommendations, and considering the unique features of recommendations in the medical field, the research framework is designed.

Most existing studies focus on data or knowledge integration, lacking deep collaboration among multi-source heterogeneous data, medical domain knowledge, and clinical needs. This limits model interpret ability and clinical applicability. In complex clinical scenarios, flexible utilization of data and knowledge is essential. Inspired by the cognitive process of physicians considering patient profiles and guidelines, we proposes a data-driven, knowledge-guided feature engineering to enhance diagnostic accuracy. Additionally, a multi-task learning algorithm based on meta-attention mechanisms is developed to prioritize relevant information, along with an automated recommendation framework mimicking cognitive management. These efforts aim to establish a dynamic data-knowledge-business model to improve disease diagnosis.

In summary, the contributions of this study are as follows:

1. Using real medical case data from Traditional Chinese Medicine, we develop a systematic feature engineering framework. This includes the design of both high- and low-order features, knowledge representation features, and various feature interaction methods. These approaches help to fully explore the information contained in the original data.
2. We propose a deep matching neural network model that integrates TCM knowledge and modifies the loss function. Furthermore, an interaction mechanism between data and knowledge is introduced, enabling the model to better understand the underlying knowledge.
3. Feature engineering and model training are automated and applied to real-world scenarios. Compared to the baseline, both offline evaluation metrics and online results show significant improvements.

## 2 Methods

**Figure 2:**
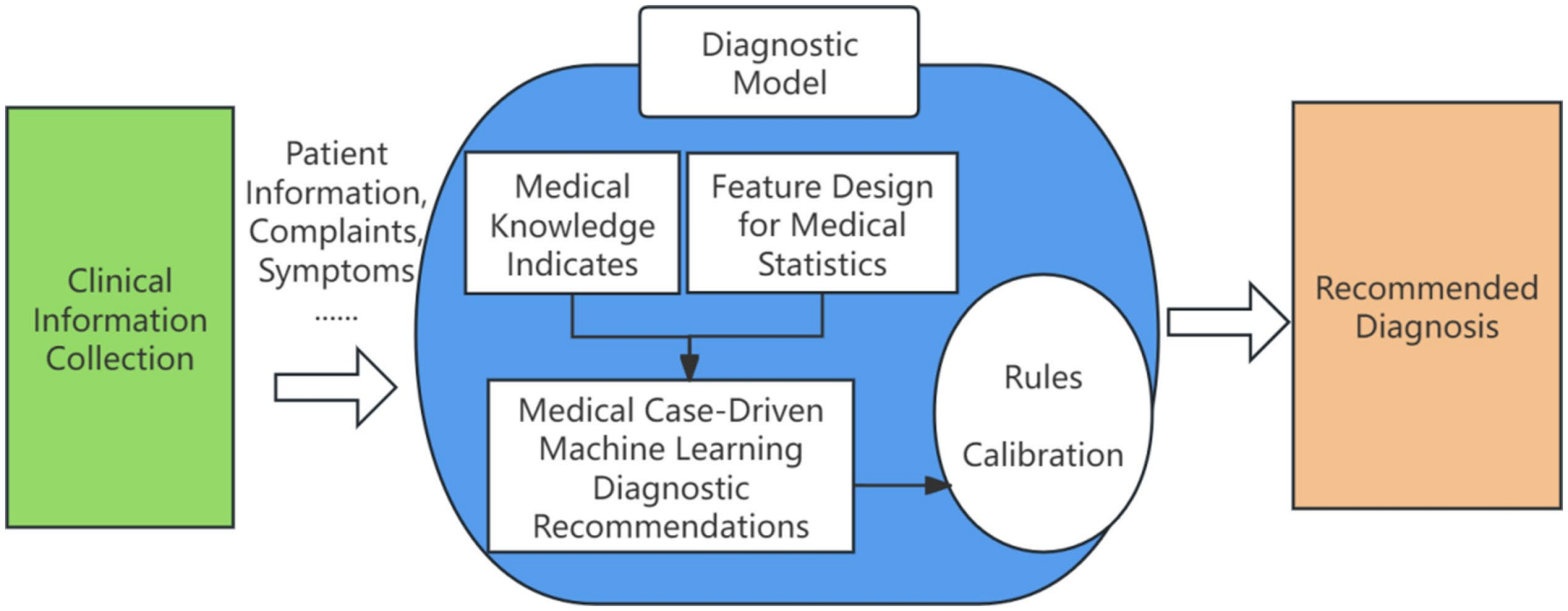
Clinical intelligence assisted diagnosis and decision recommendation. Author: Yingshuai Wang, Date: 2025-04-29.

The recommendation framework incorporates automated feature engineering and model optimization, enabling its application across a range of tasks in the medical domain. These include medical case suggestions, prescription recommendations, drug recommendations, disease diagnosis, evidence element identification, and more. An overview of the intelligent clinical decision support and recommendation in Traditional Chinese Medicine is illustrated in Figure 2.

### 2.1 Feature engineering

#### 2.1.1 Original features of Chinese medicine

This section outlines the key features derived from Traditional Chinese Medicine that serve as the foundation of the recommendation system. These include various types of information such as patient and physician details, symptoms, diagnostic findings, treatment plans, and medical case records, all of which help enhance to the accuracy and personalized recommendation.

1. Physician information: physician name, consultation department.
2. Patient information: patient name, gender, date of birth, age.
3. Objective environment: date of consultation, season of consultation.
4. Symptom information: chief complaint, history of present illness, carved symptoms, standard symptoms, source of symptoms, tongue, moss, pulse.
5. Diagnostic information: Chinese Medicine Diagnosis, Western Medicine Diagnosis, Chinese Medicine Evidence, source of evidence.
6. Treatment Information: Treatment principles and methods, formula name, formula composition, acupuncture and herbal therapy details, number of visits, and treatment results.
7. Medical case information: medical case type, medical case name, and medical case source.

#### 2.1.2 Statistical features of herbs

##### 2.1.2.1 Co-occurrence probability

The mathematical expression for co-occurrence probability is shown in Equation 1.


(1) $$P(a|b)=\frac{\text{count}(a,b)}{\text{count}(a,B)}$$

count(a,b) indicates the number of times a symptom and an evidence element appeared in the same medical case, while count(a,B) indicates the number of times a symptom and any evidence element appeared in the same medical case.

##### 2.1.2.2 Confidence

The mathematical expression of confidence level is shown in Equation 2.


(2) $$\text{confidence}(A=>B)=P(B|A)=\frac{\text{count}(A\cap B)}{\text{count}(A)}$$

count(a,b) indicates the number of times a symptom and an evidence element occurred in the same medical case in all medical cases, while count(a,B) indicates the number of times a symptom and any evidence element occurred in the same medical case in all medical cases.

##### 2.1.2.3 Degree of support

The support of an association rule A=>B indicates the ratio of the number of elements in the intersection of the item set A and the item set B to the number of elements in the total transaction set count(T) . The degree of support is used to evaluate the importance of association rules and indicates the universality of the current rule among all rules. The mathematical expression of support is shown in Equation 3.


(3) $$\text{support}(A=>B)=\frac{\text{count}(A\cap B)}{\text{count}(T)}$$

##### 2.1.2.4 TFIDF

TFIDF (Term Frequency-Inverse Document Frequency) suggests that the importance of a word is positively correlated with its frequency in a document and negatively correlated with its frequency in the whole corpus. In the task of recommending ‘evidence elements’ in Traditional Chinese Medicine (TCM), it reflects the importance of ‘evidence elements’ to ‘symptoms’ as calculated by Equation 4.


(4) $$\mathrm{TF}=\frac{n_{\mathrm{ij}}}{\sum_{k}n_{\mathrm{kj}}}$$

Where $n_{\mathrm{ij}}$ indicates the number of occurrences of the current word in the document, and $\sum_{k}n_{\mathrm{kj}}$ means the sum of the occurrences of all words in the document. The mathematical expression of Inverse Document Frequency is shown in Equation 5.


(5) $$\mathrm{IDF}=\log\frac{N}{n_{i}+1}$$

Where N denotes the total number of documents in the corpus and $n_{i}$ denotes the number of documents that contain a particular word.

#### 2.1.3 TCM domain knowledge features

TCM domain features are derived from the original features and include knowledge based on time, gender and age, Chinese and Western medicine diagnoses, and symptoms.

1. Knowledge derived based on time: year of birth, month of birth, Heavenly Stem and Earthly Branches, year calculates the five elements according to the innate weak organs, Heavenly Stem calculates the five elements of the year’s fortune, plus excess or less than the year’s fortune, the year and month of birth project the SITIAN in the spring, the main QI and politeness, and the SITIAN in the spring, the main QI and politeness to get the innate constitution.
2. Based on the knowledge derived from gender age: after the female seven male eight points, the number of years combined with gender, divided by 8 arithmetic, 001 for female 0–7 years old, 002 for female 8–14 years old, 101 for male 0–8 years old, 102 for male 9–16 years old, and so on.
3. Based on knowledge derived from Chinese and Western medicine diagnosis: This includes site labeling (e.g., acute disease = 0, chronic disease = 1), a list of disease names from TCM, and the Western medicine disease classification system.
4. Symptom-based derived knowledge: common evidence elements, common evidence element frequency, common evidence element categories (a = disease site, b = essential substance, c = disease evil, d = pathological state, e = connecting word).

### 2.2 Model design

#### 2.2.1 Meta-learning network for feature fusion

**Figure 3:**
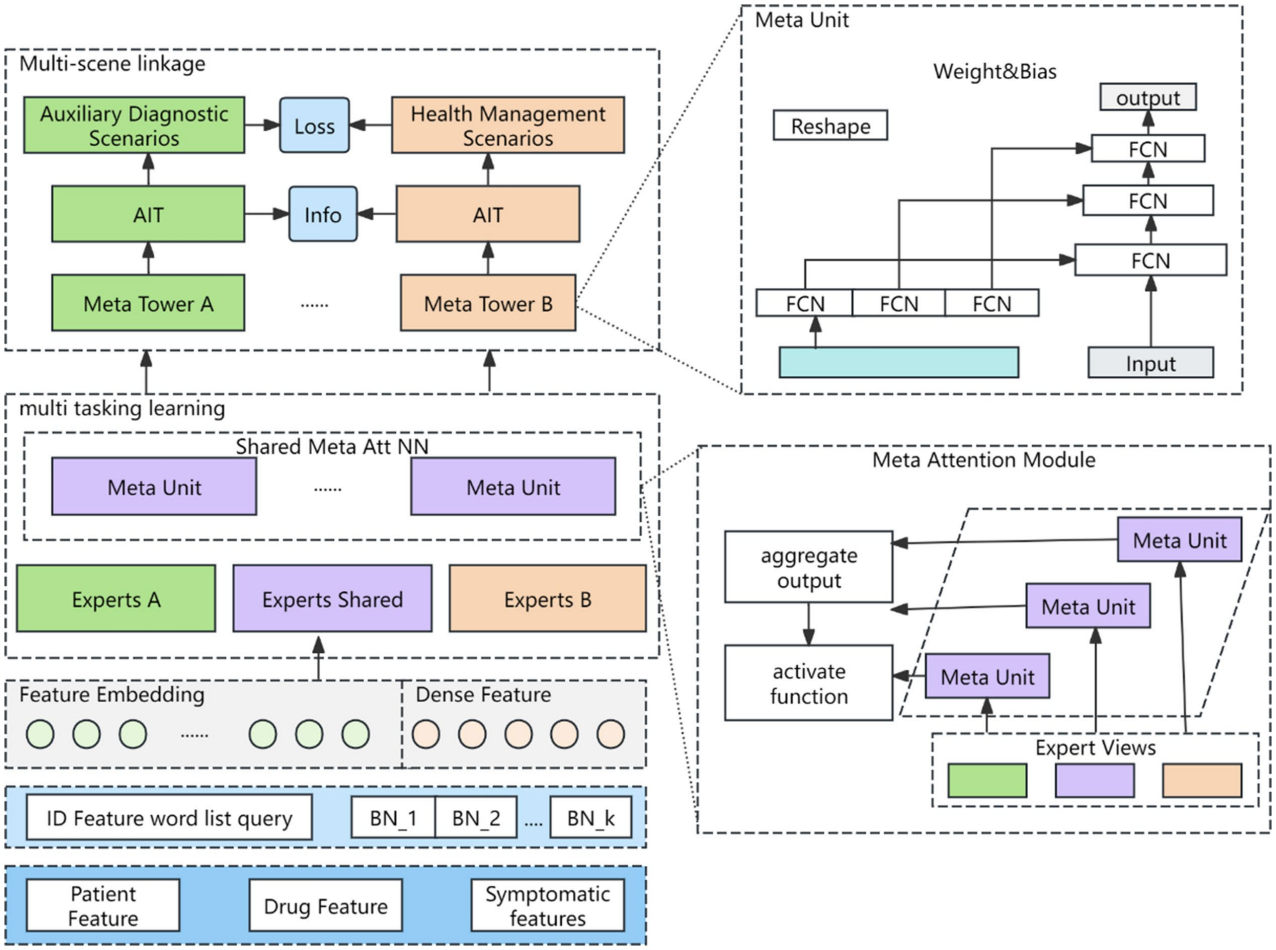
Meta-learning network. Author: Yingshuai Wang, Date: 2024-12-21.

To better capture scene-specific information, a meta-learning fine-grained attention network is introduced. This network adapts to the data distribution of various scenes via multi-objective learning, generating distinct scene representations while mitigating the impact of sample distribution variations on model performance. The structure of the meta-learning network is illustrated in Figure 3.

The features of each scenario are primarily categorized into three types: patient features, medicine features, and context features. Most of these features are sparse and high-dimensional in the CTR prediction task, and are typically transformed into low-dimensional dense vectors using embedding techniques. These vectors are randomly initialized, updated during model training, and concatenated with dense input features to form a comprehensive feature vector, as shown in Equation 6.


(6) $$e_{i}=[e_{u},e_{i},e_{s}]$$

Where $e_{u}$ means patient embedding, $e_{i}$ indicates medicine embedding, $e_{s}$ denotes scenario embedding.

The higher-order features of the patient are extracted using the self-attention mechanism with the Transformer decoder, where the decoding network comprises a Multi-head Self-Attention Network (MSA) and a Feed-Forward Network (FFN). The Self-Attention network (SAN) is implemented using Scaled Dot-Product Attention (SDPA) as defined in Equation 7.


(7) $$\text{Attention}(Q,K,V)=\text{softmax}(\frac{QK^{T}}{\sqrt{d}})V$$

Where Q,K,V represent Queries, Keys and Values respectively, d denotes the dimension of Queries, Keys and Values. We use a multi-head attention mechanism to capture the relationship between the query matrix and the key matrix from different perspectives. The mathematical expression of the multi-head attention mechanism is shown in Equation 8.


(8) $$\begin{array}{l} S=\text{Multihead}(Q,K,V)=\\ \text{Concatenate}(\mathrm{hea}d_{1},\mathrm{hea}d_{2},\ldots ,{\text{head}}_{h})W^{H} \end{array}$$

The attention expression for the i-th head is shown in Equation 9.


(9) $$\mathrm{hea}d_{i}=\text{Attention}(Q_{i},K_{i},V_{i})$$

Where $W^{H}\in R^{d*d}$ is weight matrix, h is the number of attention heads. Furthermore, this study combines FFN and MSN to enhance the model’s characterization. The key concepts are outlined in Equations 10, 11.


(10) $$\text{featur}e_{\text{high}}=\mathrm{FFN}(S)$$

(11) $$\mathrm{FFN}(x)=\text{ReLu}((xW_{1}+b_{1})W_{2})+b_{2}$$

**Figure 4:**
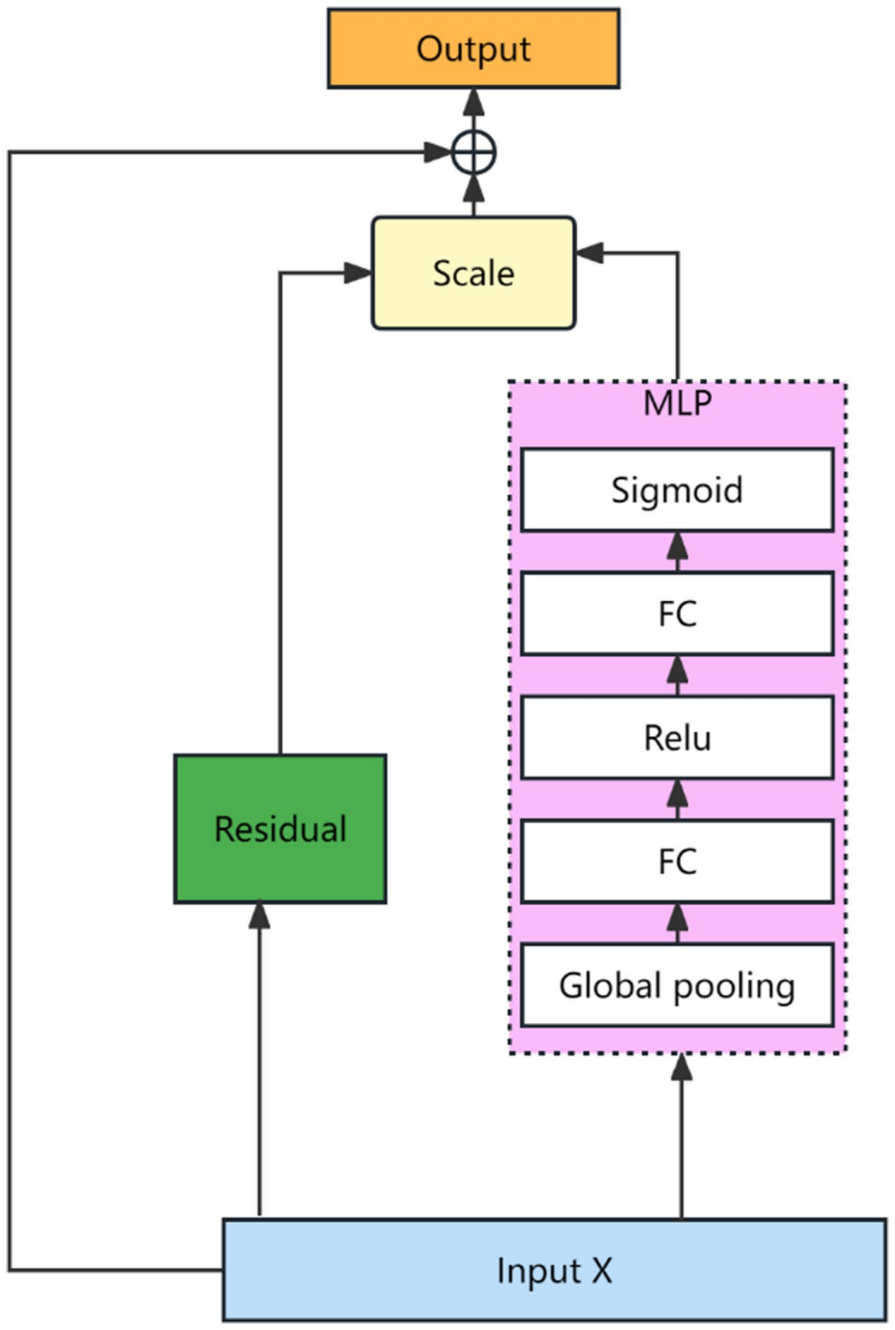
Feature adaptive fusion networks. Author: Yingshuai Wang, Date: 2024-12-21.

Where $x\in S,d_{k}=\frac{d}{h},W_{1}\in R^{d*d_{k}},W_{2}\in R^{d_{k}*d}$ , $W_{1}$ and $W_{2}$ are weight matrices, $b_{1}\in R^{d_{k}},b_{2}\in R^{d}$ , $b_{1}$ and $b_{2}$ are the bias terms. After the features are characterized as described above, they are dynamically fused using a Squeeze-and-Excitation Network (SENET) (19). The SENET structure is shown as Figure 4.

The mathematical description of the network is shown in Equations 12, 13.


(12) s=σ(MLP(z,W))

(13) $$x_{l+1}=x_{l}+s$$

Where z denotes input features, W is the weight parameter, MLP denotes a multiple layer perceptual machine and σ is a nonlinear mapping operator. The Meta Unit module is calculated as in Equations 14, 15.


(14) $$y=\mathrm{Rel}u(W_{\text{meta}}x+b_{\text{meta}})$$

(15) $$W_{\text{meta}}=\text{Reshape}(W*\text{embeddin}g_{\text{scene}}+b)$$

Where $\text{embeddin}g_{\text{scene}}$ represents the embedding of the scene type, after MLP, we get the scene-specific representation, and generate the weight and bias by reshape to form the Meta Unit of the network. The Meta Unit parameters are different in different scenes. The input is x , the output is y . The original fully connected network is y=Relu(Wx+b) , when the network training is completed, W and b are fixed, for all the samples, W and b are the same. The Meta Unit dynamically changes the W and b according to the samples. $y=\text{Relu}(W_{\text{meta}}x+b_{\text{meta}})$ , where W and b are related to the type of scene. The computational logic of Meta Attention module is shown in Equation 16.


(16) $$\alpha_{\mathrm{Ai}}=V^{T}{\text{Meta}}_{A}([E_{i}]),1\leq i\leq S+K$$

Where $\alpha_{\mathrm{Ai}}$ denotes the attention scores of the experts for task A, the expert group consists of S shared experts and K unique experts. ${\text{Meta}}_{A}$ represents the shared Meta Unit for task A. The parameters change dynamically with different scenarios, and $V^{T}$ are network learning parameters. $\alpha_{\mathrm{Ai}}$ are scalar values, and then normalized by an activation function softmax for the S + K scores. Scene information is explicitly incorporated through the attention meta-network in the multi-task attention weights, and the information of different scenes is captured when calculating the attention scores. The computational logic of the Meta Tower module is as follows: each layer of the multi-task learning tower is implemented as a fully connected network. The meta tower is constructed by cascading multiple meta units, and the parameters adapt dynamically with different scenes. This architecture enhances the model’s ability to characterize specific scenes through the layered structure of the tower.

#### 2.2.2 Automated training framework

**Figure 5:**
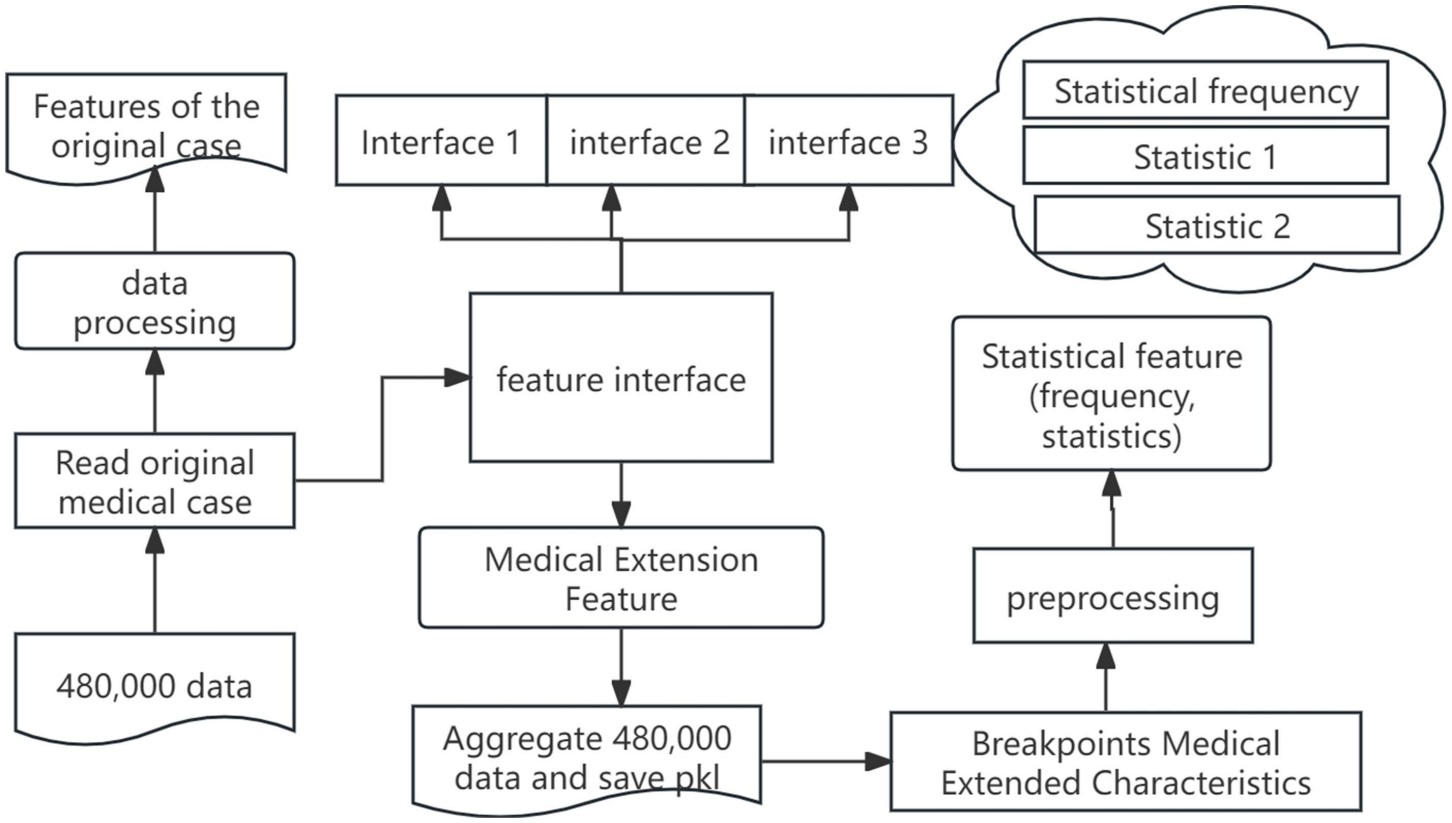
Automated feature engineering. Author: Yingshuai Wang, Date: 2024-12-21.

The automated training framework includes feature automation and recommendation model automation, and the feature automation architecture is shown in Figure 5.

The steps of feature automation are as follows: in the first stage, we perform raw-field processing by importing medical case from excel, handling missing values, and segmenting the data. Next, we extend the traditional Chinese medicine fields by transforming original symptom descriptions and integrating custom features via feature-interface. Finally, we unify all features into a standardized statistical input format, compute the necessary statistics, and serialize the extended feature set into PKL files, ensuring that the features stay continuously updated with the underlying data. The following outlines the key stages of the process, from data reading to model training, emphasizing the critical operations at each phase of the workflow.

##### 2.2.2.1 Data reading

Instantiate the data reading module and invoke the relevant functions to copy, shuffle, and sequentially retrieve the data.

##### 2.2.2.2 Model network

Firstly, abstract a general base network module. Secondly, carry out internal embedding, construct the network structure, design the loss function, and perform other related operations.

##### 2.2.2.3 Model training

Define the session graph, including the creation of the model and evaluation modules, the definition of the optimizer, and the input of score and loss into the evaluation module. Session graph computation involves tasks such as session initialization, executing sess.run() to calculate loss and evaluation metrics, and determining when to save the checkpoint.

## 3 Results

### 3.1 Experimental data

The data comes from the medical records of real patients. The following information is recorded in the case: the patient’s personal information, information about the environment at the time of consultation, information about the description of the disease, and information about the doctor’s analysis of the disease at the time of consultation and treatment. We screened 150,000 medical cases, selected 130,000 to generate the training set, and the remaining 20,000 to generate the test set. A sample example of the training data is shown in Table 1.

**Table 1:** Examples of TCM samples.

Medicine Symptoms	Syndrome	Medical elements	Elements type	Disease name
Epigastric-pain, cracked-tongue, white-coating, stringy pulse	Deficiency of vital energy, congestion of heat and toxins.	zheng, qi, kui, xu, re, du, yong, sheng	b, b, d, d, c, c, d, d	Stomach ache
Nausea, vomiting, abdominal pain, vexation, fatigue, fat tongue, yellow moss, greasy moss, fine veins	Internal obstruction by wetness	Shi, xie, nei, zu	c, c, d	Vomiting
Generalized vomiting, excessive salivation, pale mouth, pale red tongue, white moss, sluggish pulse	Rebellious cold in the stomach	Wei, han, shang, ni	a, c, a, d	Vomiting
Weakness, Yellow urine, Stringy pulse	Stagnant heat in the liver meridian, blood stasis	gan jing, yu, re, yu	a, d, c, c	Liver accumulation
Tightness in chest, palpitation, shortness of breath, poor appetite, poor sleep, dry stools, dark red tongue, scanty coating	Double deficiency of the heart and spleen	xue, xin, xu, qi, pi	b, a, d, b, a	Tachycardia
Eye pain, itchy eyes, red tongue, thin moss, white moss, fine pulse, stringy pulse	Deficiency of liver and kidney	gan, xu, shen	a, d, a	Cataract

Author: Yingshuai Wang, Date: 2024-12-21.

### 3.2 Comparison of methods

Text-CNN (20): Text-CNN is a multi-label framework that leverages convolution neural networks to construct model architectures.

MLP (15): Multiple layer Perceptron Machine is a baseline for deep learning and is widely used in recommendation systems.

MMOE (18): A model based on multi-task expert sharing, which can flexibly adjust the weights of experts for different tasks through gated networks.

Our model: The model proposed by us based on feature fusion of multi-task learning and meta-learning network, which can interact the information from different scenarios.

### 3.3 Evaluation metrics

The evaluation metrics used in this research are AUC, MR, MRR, Hits@10, and Unit Hit Rate.

1. AUC a metric used to measure the effectiveness of the ranking prediction model, the closer the value is to 1, the better the model is, mathematically defined as in Equation 17.

(17) $$\mathrm{AUC}=\frac{1}{|T^{+}\|T^{-}|}\sum_{x^{+}\in T^{+}}\sum_{x^{-}\in T^{-}}[g(x^{+})>g(x^{-})]$$

Where $T^{+}$ , $T^{-}$ denote positive and negative sample sets, $|T^{+}|$ , $|T^{-}|$ denote the number of positive sample and negative sample g(x) is the model predictive function, I(x) is an indicative function.

1. Hits@10 denotes the probability of hitting the real labels, which are ranked in the top 10 predicted objects by the model output, the higher the better, as defined in Equation 18.

(18) $$\text{Hits}@10=\frac{\text{label}\cap \text{pred}\_10}{\text{numbers of label}}$$

Where pred_10 denotes the top 10 ranked evidence elements predicted by the model.

1. MR (Mean Rank) is used to measure the likelihood of the model incorporating errors, the smaller the better, defined as in Equation 19.

(19) $$\mathrm{MR}=\frac{1}{|S|}\sum_{i=1}^{|S|}{\mathrm{rank}}_{i}=\frac{1}{|S|}(\mathrm{ran}k_{1}+\mathrm{ran}k_{2}+\ldots +\mathrm{ran}k_{|S|})$$

1. MRR (Mean Reciprocal Ranking) reflects the generalization ability and robustness of the model, with higher values indicating better performance. The mathematical expression of MRR is shown in Equation 20.

(20) $$\mathrm{MRR}=\frac{1}{|S|}\sum_{i=1}^{|S|}\frac{1}{{\mathrm{rank}}_{i}}=\frac{1}{|S|}(\frac{1}{\mathrm{ran}k_{1}}+\frac{1}{\mathrm{ran}k_{2}}+\ldots +\frac{1}{\mathrm{ran}k_{|S|}})$$

### 3.4 Experimental setup

The TCM recommendation model is implemented based on the TensorFlow framework, and one GPU (Tesla V100-PCIE-32GB) is used for training and testing. In order to be comparable between models, the data set and hyper-parameters are maintained the same. In order to prevent the interference of indicator fluctuation on the results, the models were trained five times for each experiment, and the evaluation indicators were averaged for each model.

### 3.5 Experimental results

The TCM evidence elements are divided into five categories: disease location, essence substance, disease evil, pathological state and association relationship, in order to enhance the learning ability of the model, the evidence elements are split according to the categories they belong to, and are viewed as five tasks for modelling. Table 2 shows the performance of each model in different datasets.

**Table 2:** Multi-task meta-learning model effects.

Model	Hits@10	*p* value	MeanRank	*p* value	MRR	*p* value	AUC	*p* value
MLP	0.76644	base	3.00478	base	0.33280	base	0.90844	base
TextCNN	0.77080	0.1987	2.95720	0.4029	0.33812	0.5872	0.90991	0.2583
MMOE	0.76824	0.2018	2.98508	0.3964	0.33500	0.0973	0.90870	0.1476
Our model	**0.78713**	**0.0392**	**2.94366**	**0.0417**	**0.33959**	**0.0358**	**0.92974**	**0.0485**

Author: Yingshuai Wang, Date: 2025-04-27.

The bold values in Table 2 represent the optimal performance for each evaluation metric in the model comparison experiments.

In the TCM evidence element recommendation task, there is a clear difference from the traditional e-commerce recommendation task. In e-commerce recommendation, there is no distinction between correct and incorrect products, while each item in a TCM medical case corresponds to an exact evidence element. This feature makes the evaluation of recommendation results more concerned with the accuracy of the first 10 evidence elements rather than the global comprehensive evaluation of recommendations. We introduce a custom metric called unit hit rate. By emphasizing the top-ranked correct evidence elements, this metric provides a more intuitive assessment of the model’s recommendation performance in the TCM domain. The evaluation metric not only focuses on the accuracy of the recommendation results, but also on their importance and effectiveness in practical applications. The unit hit rate is calculated as Equation 21.


(21) $$\mathrm{Hit}s^{k}=\frac{1}{N}\sum_{i=1}^{N}n_{i}\;\;\;n_{i}=\{\begin{array}{c} 1\;\;\text{if}\;\mathrm{pre}d_{i}^{k}\;{\text{in label}}_{i}\\ 0\;\;\;\;\text{otherwise} \end{array}$$

**Figure 6:**
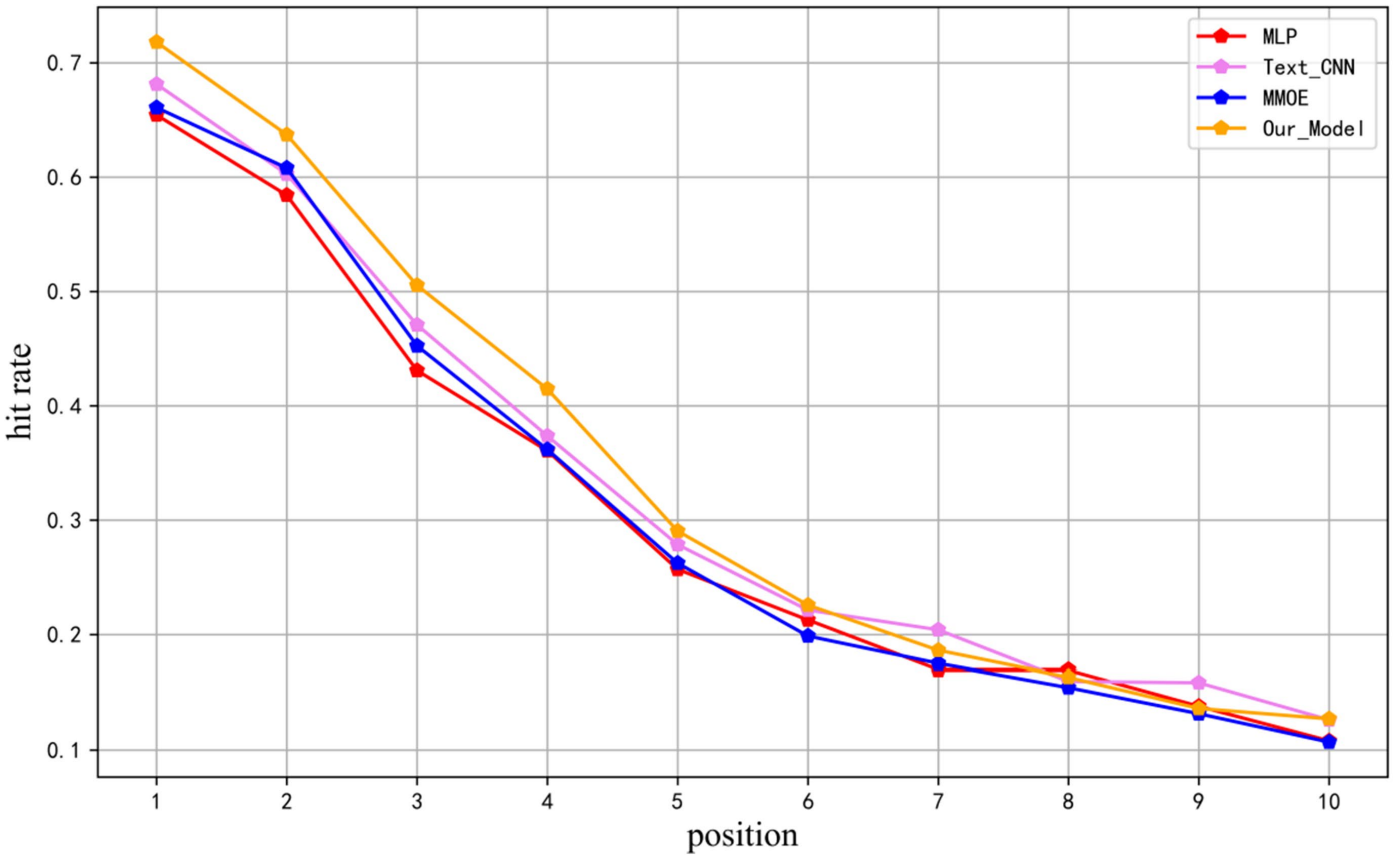
Unit hit rate. Author: Yingshuai Wang, Date: 2024-12-21.

Where k denotes the position of the predicted evidence elements after sorting, N represents the number of samples, $n_{i}$ denotes the correct evidence of the sample, and $\mathrm{pre}d_{i}^{k}$ denotes the predicted evidence at the kth position for the ith sample. This evaluation metric is calculated by dividing the number of correct evidence elements at the kth position in the prediction by the total number of samples, providing insight into the distribution of correct evidence elements within the prediction results. The metric was computed for the top 10 positions in the test results of MLP, TEXTCNN, MMOE, and the models presented in this study, as illustrated in Figure 6.

It can be seen that the models proposed in this research substantially outperform baselines in the first six positions, with an average number of correct evidence elements in the test samples of 4.489, and the distributions of correct evidence elements in the top 10 recommended results are all ahead of other models.

## 4 Discussion

### 4.1 Advanced characteristics of the model

**Figure 7:**
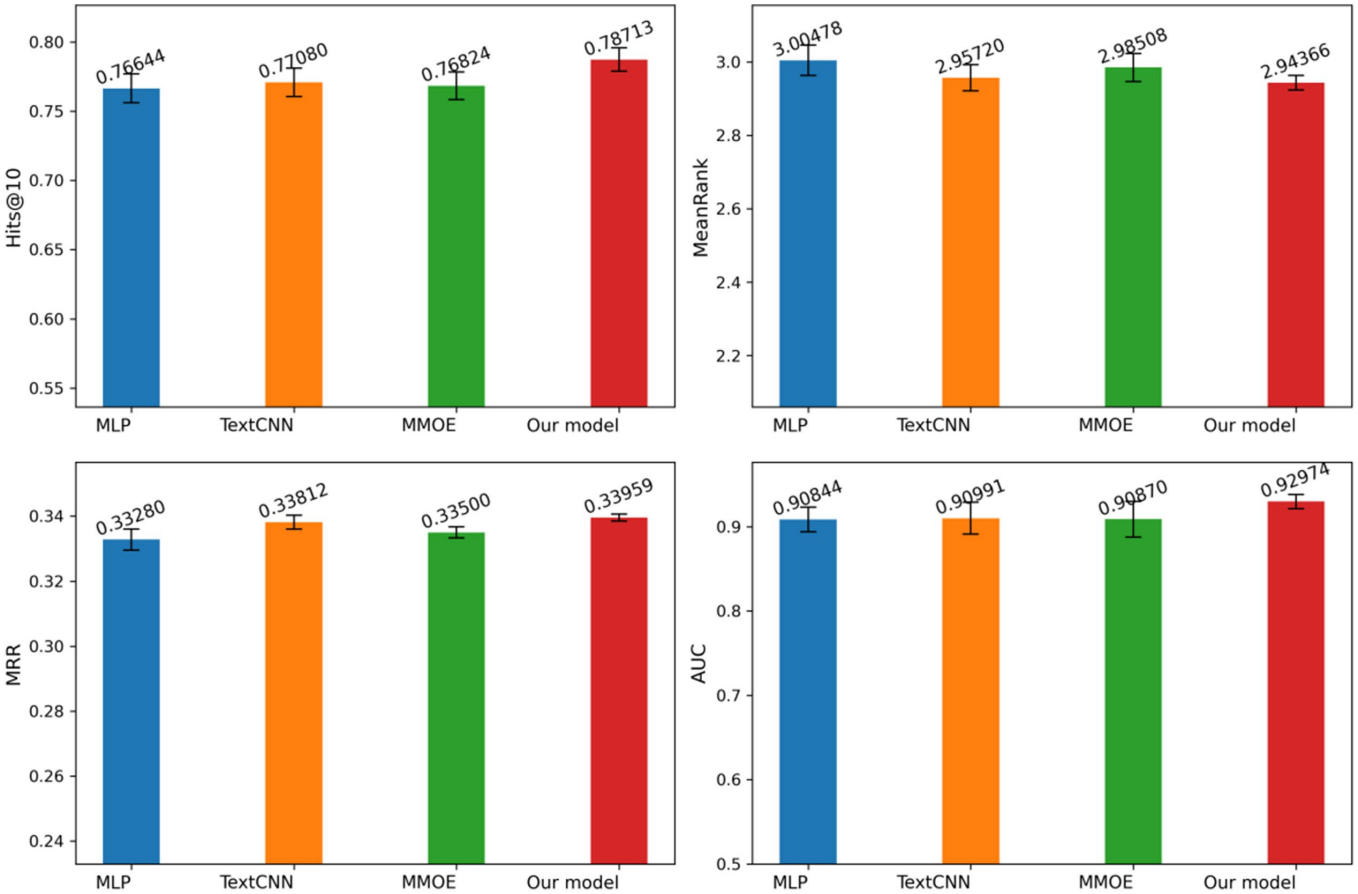
Evaluation metrics and standard deviations for different models. Author: Yingshuai Wang, Date: 2025-04-29.

Figure 7 illustrates our model performance compared to MLP, TextCNN, and MMOE across Hits@10, MeanRank, MRR, and AUC metrics, with standard deviation represented by error bars. Our approach consistently outperforms all baselines across these metrics while maintaining minimal variance. The results demonstrate our model superior precision and remarkable stability. This comprehensive evaluation provides compelling evidence of our method effectiveness and advantages over existing approaches.

### 4.2 Discussion of the ablation experiment

To more effectively compare the impacts of different enhancement points in the model, we conducted four ablation studies. These experiments focused on herbal statistical features (Test1), traditional Chinese medicine domain knowledge features (Test2), the meta-attention unit (Test3), and the feature adaptive fusion network (Test4). The results of the ablation studies are illustrated in Table 3.

**Table 3:** Ablation study results.

AB test	Hits@10	*p* value	MeanRank	*p* value	MRR	*p* value	AUC	*p* value
Test1	0.76975	0.0478	2.95832	0.0492	0.33780	0.0563	0.91042	0.0573
Test2	0.77482	0.0498	2.96120	0.0531	0.33742	0.0481	0.91875	0.0493
Test3	0.77591	0.0378	2.95609	0.0467	0.33805	0.0479	0.90998	0.0462
Test4	**0.77307**	**0.0362**	**2.94366**	**0.0403**	**0.33959**	**0.0329**	**0.92974**	**0.0438**

Author: Yingshuai Wang, Date: 2025-04-27.

The bold values in Table 3 indicate the best performance for each evaluation metric in the ablation experiments.

### 4.3 Discussion of the unit hit rate behavior

Unit Hit Rate behavior is a novel metric introduced in this study, specifically designed for evaluating models in the medical domain. It emphasizes the precision of the top-ranked items in the model’s recommendation list. Our model performs the best across all metrics, achieving 0.7176 in hits@1, 0.6371 in hits@2, 0.5054 in hits@3, and 0.6201 in Hits_avg, which is the average of the first three positions, significantly outperforming other models. With a *p* value of 0.0279, the improvement is statistically significant compared to the baseline model, as shown in Table 4.

**Table 4:** Unit hit rate behavior.

Model	hits@1	hits@2	hits@3	Hits_avg	*p* value
MLP	0.6544	0.5842	0.4309	0.5565	base
Test_CNN	0.6814	0.6026	0.4708	0.5850	0.0973
MMOE	0.6609	0.6080	0.4525	0.5738	0.1856
Our model	0.7176	0.6371	0.5054	0.6201	0.0279

Author: Yingshuai Wang, Date: 2025-04-25.

The bold values in Table 4 indicate the best performance for the ‘unit hits behavior’ evaluation metrics.

## 5 Conclusion

In this article, a knowledge-driven data scenario modelling approach is proposed. A representation based on association rules and domain knowledge is adopted, and the knowledge representation is transformed into mathematical vectors, which not only captures the relevance in the domain, but also provides more accurate information for the model. A data knowledge fusion neural network is proposed, which improves the model’s understanding of knowledge by constructing auxiliary tasks and designing the interaction function between the main task and auxiliary tasks. A unit hit rate evaluation index is proposed to focus on the accuracy of the prediction of the forward position, which better measures the recommendation prediction effect of the model in the field of traditional Chinese medicine, and provides a more targeted direction for the iteration of the model. An automated framework for feature engineering and recommendation algorithm model training is designed and applied to a real TCM medical case evidence prediction task, demonstrating the model’s effectiveness, which has been recognized by experts in the TCM field.

Although our approach has achieved significant results in TCM syndrome prediction, its association rules and knowledge representation depend on TCM specific theoretical systems, potentially limiting adaptability when transferred to Western medicine. While auxiliary task design enhances the model’s perception of domain knowledge, the decision-making processes within deep neural networks still lack interpret ability, potentially undermining clinical expert trust in system outputs. Additionally, current evaluations primarily rely on historical case back-testing and offline metrics, without prospective clinical pilots or user experience studies, making it difficult to fully reflect the model’s utility in real diagnostic scenarios. Future research will focus on developing generalized data-knowledge fusion algorithms, incorporating explainable techniques, advancing clinical application pilots, and establishing compliant data sharing or federated learning platforms to achieve greater practicality and universality.

## Data Availability

The original contributions presented in the study are included in the article/supplementary material, further inquiries can be directed to the corresponding author.
